# Effect of Consumption of Cocoa-Derived Products on Uric Acid Crystallization in Urine of Healthy Volunteers

**DOI:** 10.3390/nu10101516

**Published:** 2018-10-16

**Authors:** Antonia Costa-Bauza, Felix Grases, Paula Calvó, Adrian Rodriguez, Rafael M. Prieto

**Affiliations:** Laboratory of Renal Lithiasis Research, University Institute of Health Sciences Research (IUNICS-IdISBa), University of Balearic Islands, Ctra. Valldemossa km 7.5, 07122 Palma de Mallorca, Spain; antonia.costa@uib.es (A.C.-B.); pauletaa.95@hotmail.com (P.C.); adrian.rodriguez@uib.es (A.R.); rafelm.prieto@uib.es (R.M.P.)

**Keywords:** uric acid, urolithiasis, cocoa, theobromine, therapy

## Abstract

The purpose of this study was to determine the effects of consumption of different cocoa-derived products on uric acid crystallization in urine of 20 healthy volunteers. Participants were requested to select the specific diet that they wished to follow during the 12 h prior to collection of urine. The only restriction was that the diet could not include any product with cocoa, coffee, or caffeine. On the first day, each volunteer followed their selected diet, and an overnight 12 h urine sample was collected as the baseline urine. After seven days on an unrestricted diet, each volunteer repeated the same diet with 20 g of milk chocolate, chocolate powder, or dark chocolate during breakfast and another 20 g during dinner. Overnight 12 h urine samples were then collected. Urine volume, pH, oxalate, creatinine, uric acid, theobromine, and a uric acid crystallization test were determined for each sample. The results for all 20 patients show that uric acid crystallization was significantly lower following the consumption of chocolate powder or dark chocolate relative to baseline or following the consumption of milk chocolate. The results indicated that increased concentrations of urinary theobromine reduced the risk of uric acid crystallization.

## 1. Introduction

Renal lithiasis currently has a prevalence of about 10% worldwide [1], and the estimated prevalence will be about 30% by 2050 [2]. Renal calculi are composed of different substances, and uric acid is present in 7 to 14% of all calculi [3,4,5,6]. Uric acid stones mainly affect males, have a high rate of recurrence, and are associated with other pathologies, such as diabetes and obesity [7,8,9]. In general, 50 to 70% of patients with renal lithiasis who do not receive treatment or do not modify their diets will develop new renal calculi within five years [10,11], while very variable recurrence rates (5 to 100% per year) have been observed for patients with calcium kidney stones who modify their diet or follow a pharmacological treatment [12].

The main cause of uric acid crystallization is the supersaturation of urine [13], although there are other important factors. For example, urinary pH may be more important than hyperuricosuria, because a pH below 5.5 markedly decreases the solubility of uric acid. In fact, patients with uric acid stones have lower urinary pH than healthy individuals [14,15]. On the other hand, many healthy individuals have urinary pH values below 5.5 and high uric acid concentration, but no uric acid stones. Consequently, a low pH contributes to the formation of uric acid kidney stones, but there are other factors.

The dietary measures and pharmacological treatments currently used to treat uric acid stones have not changed very much in the last 25 years. These recommendations mainly consist of reduced consumption of purine-containing foods, increased fluid intake, urinary alkalization, and administration of allopurinol or febuxostat, but their use should be limited to patients with high urinary uric acid levels and calcium stones or for patients in which alkalinization is not successful [16,17,18]. None of the strategies for treatment or prevention of uric lithiasis employ crystallization inhibitors, and there are no medically available inhibitors of uric acid crystallization.

Recent research indicated that theobromine can inhibit uric acid crystallization, suggesting it may be useful for the prevention of uric acid urolithiasis [19]. Theobromine is, thus, the first natural product known to inhibit the crystallization of uric acid, and has potential for use in the treatment of renal stone formers. Moreover, theobromine is excreted in urine at concentrations similar to those needed to prevent uric acid crystallization [20,21].

Theobromine is a dimethylxanthine that is abundant in cocoa and cocoa-derived products, such as chocolate [22]. Clinical studies indicated that its half-life in serum is 6.1 to 10 h, and that 16 to 18% of a single dose of 10 mg/kg is excreted unchanged after 48 h [23,24]. Consumption of caffeine can also lead to excretion of theobromine, and about 11% of an oral dose of caffeine is excreted as theobromine due to metabolism in the liver [25].

In this paper, we developed a method to assess the urinary uric acid crystallization risk (UAC test), and then used this test to determine the effects of consumption of different cocoa-derived products on the capacity of the urine of healthy subjects to form uric acid crystals.

## 2. Materials and Methods

### 2.1. Chemicals and Reagents

Uric acid, theobromine, and acetonitrile, which were all high performance liquid chromatography (HPLC) gradient grade, were from Sigma (St. Louis, MO, USA). All solutions were prepared in ultra-pure water from a Milli-Q water purifier. Analytical and guard columns were from Phenomenex (Torrance, CA, USA).

### 2.2. Participants

Twenty healthy adult volunteers (11 males and 9 females, mean age: 37 years, age range: 22 to 65 years) from Mallorca, Spain, participated in the study. None of the participants had chronic diseases or were receiving pharmacological treatments, and none were allergic to theobromine or cocoa/chocolate. All subjects provided written informed consent. The nutritional intervention protocol (IB 3475/17 PI) was approved by the local Ethics Investigation Committee of the Balearic Islands, Spain. The authors (A.C.-B., F.G., A.R., R.M.P.) declare that they are the inventors of a patent based on theobromine capacity as crystallization inhibitor of the formation of crystals of uric acid in urine (International Application No.: PCT/ES2015/070301).

### 2.3. Nutritional Intervention and Urine Collection 

Participants were requested to select the specific diet that they wished to follow during the 12 h prior to collection of every urine, with the aim of reducing the influence of diet on the urinary composition as much as possible. The only restriction was that the diet could not include any product with cocoa, coffee, or caffeine.

On the first day, each volunteer followed his or her selected diet, and an overnight 12 h urine sample (starting at 8:00 p.m. and ending with the first morning urine at 8:00 a.m.), was collected and designated as the baseline urine (B).

After 7 days on an unrestricted diet, each volunteer repeated the same diet with 20 g of milk chocolate, chocolate powder, or dark chocolate during breakfast and another 20 g during dinner. Overnight 12 h urine samples were collected after 7 days of each cocoa product consumption. These cocoa-derived products were all from the same commercial source.

### 2.4. Sample Analysis

Urine volume, pH, oxalate, creatinine, uric acid, theobromine, and the UAC test were determined for each sample. Urinary pH was measured with a Crison pH 25, oxalate was determined enzymatically by the oxalate oxidase/peroxidase method (LTS, Milano, Italy), creatinine by the kinetic Jaffe method, uric acid by the uricase method, and theobromine by HPLC, as described previously [20]. The HPLC system (Waters, Milford, MA, USA) had an automatic injector (WISP700), a pump system (600), a photodiode array detector (PDA 996), and Empower software. The analytical column was a 5-μm reversed-phase column (Gemini C18 110 A; 150 × 4.6 mm) protected with a Phenomenex security C18 guard cartridge (4 × 3.0 mm). Mobile phase A was ammonium acetate (20 mmol/L, pH 7.5): acetonitrile (98:2, *vol*/*vol*) and mobile phase B was pure acetonitrile. The system was programmed in steps of 0–18% B over 15 min, maintenance of 18% B for 3 min, return to the initial conditions over 1 min, and equilibration for 4 min. The mobile phase was continuously sparged with nitrogen. The injection volume was 10 μL, and theobromine was eluted at a flow rate of 1 mL/min. Peak areas were measured at 273 nm.

### 2.5. UAC Test

**Figure 1:**
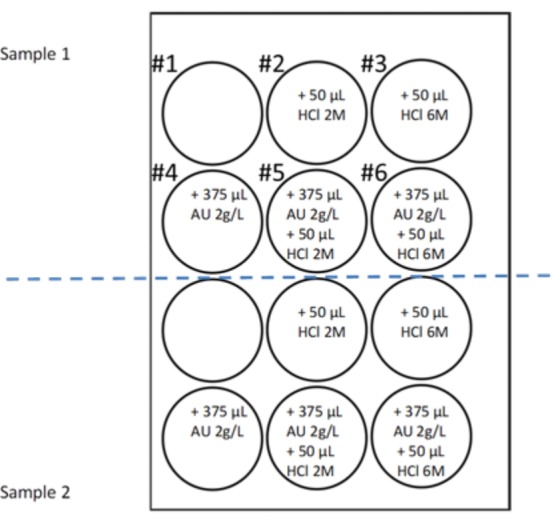
Uric acid crystallization (UAC) test. Six wells of a 12-well plate were used for each sample of urine, with each well containing 5 mL of urine and different amounts of HCL and uric acid. Well #1 had urine alone, well #2 had urine and 50 μL of 2 M HCl (0.1 mmoL H^+^), well #3 had urine and 50 μL of 6 M HCl (0.3 mmol H^+^), well #4 had urine and 750 µg uric acid (375 µL of 2 g/L solution), well #5 had urine, 50 μL of 2 M HCl and 750 µg uric acid, and well #6 had urine, 50 mL 6 M HCl and 750 µg uric acid. Uric acid (AU). + means addition of the specified volume of the solution.

The UAC test (Figure 1) was performed in polystyrene non-treated 12-well plates (Corning, NY, USA). First, 5 mL of each urine sample was added to each of 6 wells. The first well had no additions, but hydrochloric acid and/or uric acid was added to the other wells to promote the crystallization of uric acid. The 6 wells were kept at room temperature for 24 h, after which the urine was removed. The UAC test of each sample was determined by the number of wells in which there were uric acid crystals, ranging from 0 to 6).

### 2.6. Statistical Analysis

The normality of the data was assessed by plotting histograms and using the Shapiro-Wilk test. For continuous variables, all results are expressed as means ± standard deviations or as medians and percentages. A repeated measures ANOVA, with a Bonferroni post hoc test for normally distributed variables, was used. Alternatively, the non-parametric Wilcoxon signed-rank test was used to assess differences between groups. A *p* value below 0.05 was considered statistically significant. IBM SPSS Statistics version 22^®^ for Windows (SPSS Inc., Chicago, IL, USA) was used for statistical analyses.

## 3. Results

**Figure 2:**
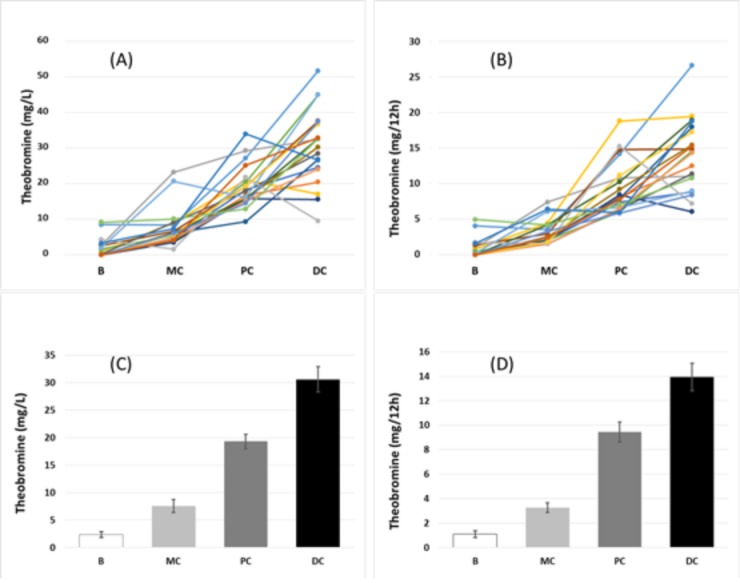
Effect of the consumption of different cocoa products on urinary theobromine concentration (**A**) and total theobromine excretion in 12 h (**B**) in each of the 20 volunteers (each colored line corresponds to one of the volunteers), and mean concentration (**C**) and total excretion in 12 h (**D**) among all 20 volunteers. An ANOVA repeated measures test indicated significant differences among the four groups in concentration and excretion (*p* < 0.05). B: basal, MC: milk chocolate, PC: chocolate powder, and DC: dark chocolate.

Figure 2A shows the urinary concentration of theobromine and Figure 2B shows the total theobromine excretion in 12 h in each of the 20 volunteers. Pooling these data (Figure 2C,D) indicated that the mean urinary concentration of theobromine and total theobromine excretion in 12 h was significantly greater after consumption of each cocoa-derived product than at baseline (*p* < 0.05 for each comparison).

Table 1 shows that the different urine samples had no significant differences in pH or uric acid. However, the urinary oxalate concentration was significantly greater following consumption of dark chocolate than at baseline, or following consumption of chocolate powder (*p* < 0.05 for each comparison).

The reproducibility of the UAC test has been studied, using the urine of two different healthy volunteers, repeating the test five times for each urine. As can be seen in Table 2, an absolute reproducibility of the UAC test results was observed, which justifies the validity of the test.

**Figure 3:**
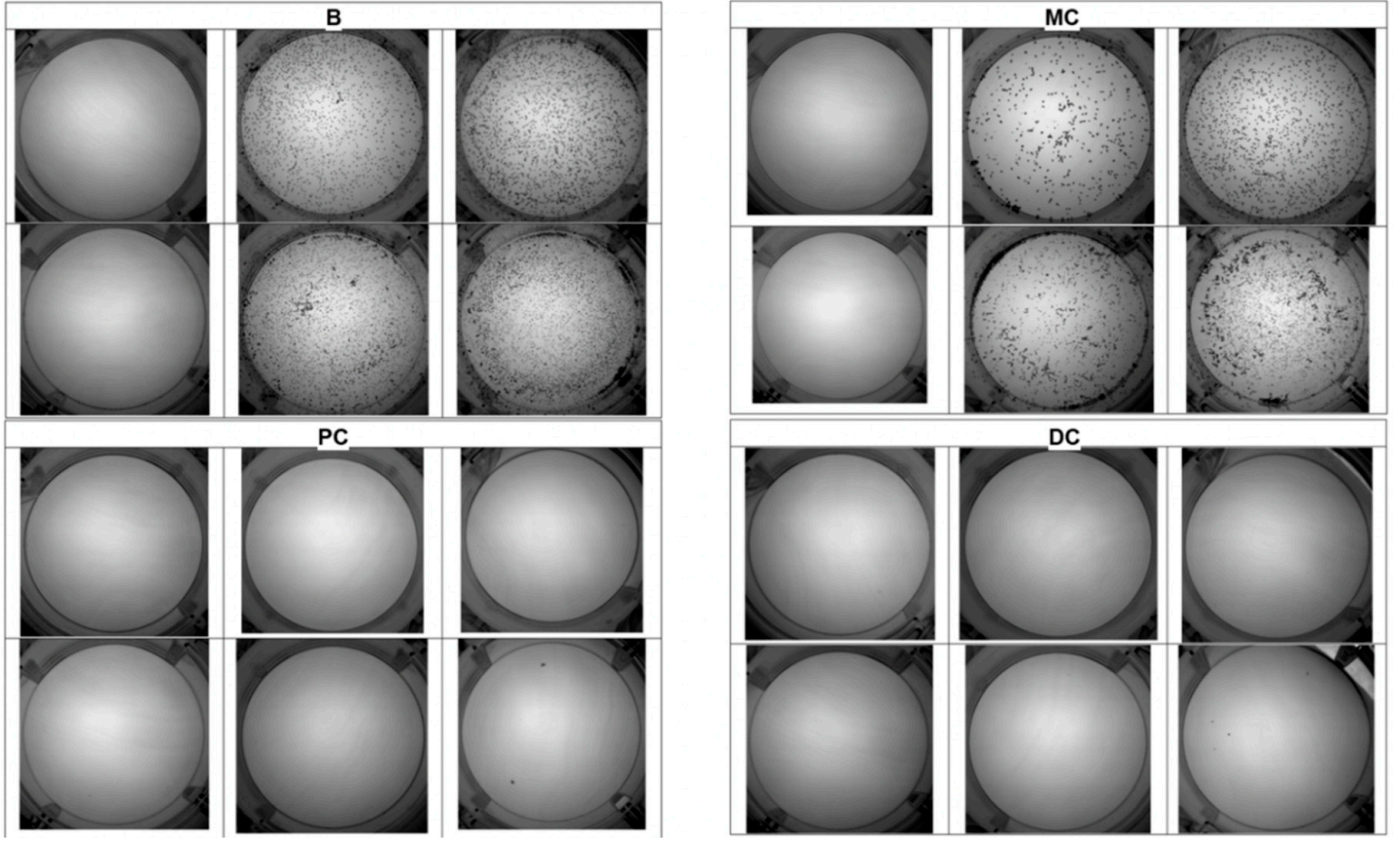
Representative UAC test results of one volunteer at baseline (B) and after consumption of milk chocolate (MC), chocolate powder (PC), and dark chocolate (DC). Uric acid crystallization was greatly reduced following consumption of chocolate powder or dark chocolate.

**Figure 4:**
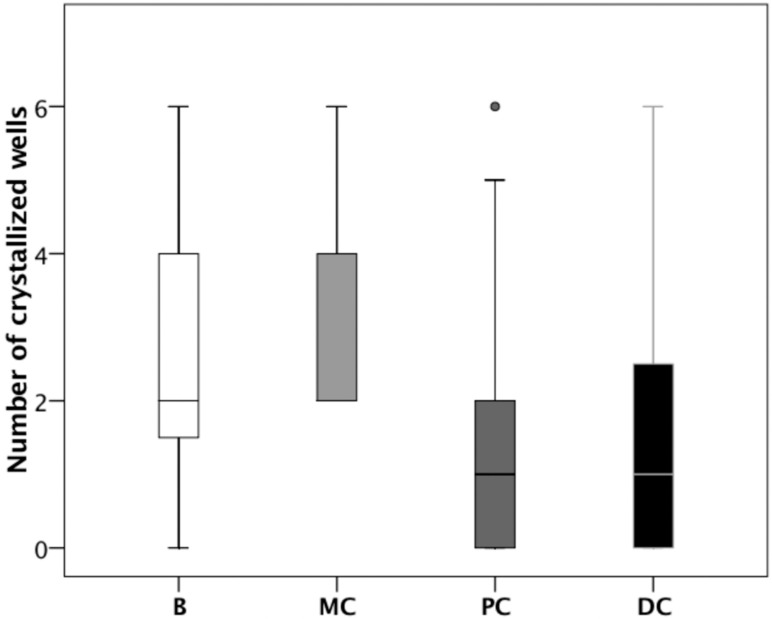
UAC test results at baseline (B) and after consumption of milk chocolate (MC), chocolate powder (PC), and dark chocolate (DC). Samples were compared using the Wilcoxon signed-rank test. Boxplots show the median (inner horizontal line), interquartile range (box), 95% confidence interval (vertical lines), and outlier (circle).

We used the UAC test to examine urine samples at baseline and after consumption of the different cocoa-derived products. Figure 3 shows the results of one representative individual. This individual had markedly reduced crystallization following the consumption of chocolate powder (PC), and dark chocolate (DC). The results for all 20 patients show that the UAC was significantly lower following the consumption of chocolate powder (PC) or dark chocolate (DC) relative to baseline (B), or following consumption of milk chocolate (MC). In Figure 4, *p* < 0.05 for each comparison).

## 4. Discussion

Uric acid renal lithiasis affects many individuals, and stone removal does not resolve the underlying pathological conditions. Thus, if the metabolic alterations responsible for stone formation are not corrected, there is a risk of future calculi [27]. As with other substances that precipitate in urine, the driving force is the supersaturation of a solute, so one might naively think that simply reducing supersaturation will solve the problem. Nevertheless, there are situations in which this is difficult to achieve, or requires great effort and willpower by the patient. For example, patients with metabolic syndrome have high urinary uric acid concentrations, and a urinary pH below 5.5, making them especially predisposed for uric acid kidney stones [28]. The current treatments thus require modification of the diet, with reduced consumption of purine-containing foods, and administration of urinary alkalinizers or xanthine oxidase inhibitors to reduce uric acid supersaturation. High patient compliance is necessary. However, pharmacological treatments can lead to certain long-term adverse effects, and patient adherence can be difficult. Moreover, the chronic consumption of alkalinizers can increase the urinary pH to above 6.2, and this can favor phosphatic lithiasis [29]. Therefore, alternative treatments are needed for uric acid renal lithiasis.

One possible strategy is the use of crystallization inhibitors. These inhibitors must be safe, easily administered, absorbed through the gastrointestinal tract, and mostly eliminated in the urine, to achieve clinically effective concentrations. For uric acid renal lithiasis, no medically applicable substance had these properties, until the recent findings of the effects of theobromine [19]. Our results indicate that theobromine meets all the necessary requirements for treatment of uric acid stones. In particular, consumption of cocoa-derived products (milk chocolate, chocolate powder, and dark chocolate), which contain different amounts of theobromine, led to increased excretion and concentration of urinary theobromine (Figure 2), and this increase was greater for chocolate powder and dark chocolate than milk chocolate, which has a lower theobromine content. Moreover, the urinary concentrations of theobromine that we achieved were similar to those that inhibited crystallization in vitro [19].

To determine whether the presence of urinary theobromine prevents the formation of uric acid crystals, we performed UAC tests for urine samples collected at baseline (basal conditions) and after the consumption of different cocoa-derived products. The UAC test was designed so that in basal conditions crystallization of uric acid would occur in at least one of the wells when incubated at room temperature for 24 h (Figure 1), since in the absence of crystallization, the inhibitory effects are not manifested. In particular, each sample was subjected to different levels of acidification (by the addition of HCl) and/or an increase of uric acid (by addition of a uric acid solution), so that the uric acid concentration was 150 mg/L higher (Figure 1). Thus, in one of the wells, an aliquot of the urine to be evaluated was introduced without any type of additive. It is evident that crystallization of uric acid depends on the pH and uric acid concentration of this urine and, as above commented, without uric acid crystallization it is not possible to evaluate the inhibitory effects of theobromine. For this reason aliquots of the same sample were added into two wells, in which the pH was decreased by the addition of two different amounts of HCl. Although the pH values reached were much lower than the physiological values (4.7 ± 0.1 and 2.8 ± 0.3), they favored uric acid crystallization and allowed us to evaluate whether the increase in inhibitor concentration (theobromine) manifested inhibitory effects, since the urine of the same individual was compared before and after the intake of this substance. It is also possible that due to the low concentrations of uric acid in the native urine, its crystallization will not occur. For this reason, another aliquot of the sample was added into a well in which a supplement of uric acid (0.75 mg) was added. Finally, to ensure that at least one well in which uric acid crystallization occurs was available, two wells with sample aliquots were added with the two HCl supplements already used together with the addition of 0.75 mg of uric acid. In this way, we ensured that we could assess the majority of situations if an increase in the concentration of urinary theobromine for a given individual increases the capacity of inhibition of uric acid in its urine.

Interestingly, only 2 of the 20 healthy volunteers presented with uric acid crystals in untreated urine, indicating that 90% of them had no risk of uric acid crystallization.

The UAC test value of each sample ranged from 0 to 6, based on the number of wells with visible uric acid crystals (Figure 3). We also used other methods to assess the UAC test, such as an estimation of the percentage area of the crystals relative to the total area of the well, but this was very time consuming and did not improve our estimates of risk. We ultimately chose the simplest methodology.

Our UAC test results indicated that a greater concentration of theobromine reduced the risk of uric acid crystallization (Figure 4). In particular, the consumption of chocolate powder and dark chocolate both clearly decreased uric acid crystallization, although milk chocolate did not have a significant effect. This is consistent with the lower content of theobromine in milk chocolate and the lower urinary theobromine concentration of individuals following consumption of milk chocolate.

Although our study is the first evidence of the in vivo effects of theobromine as an inhibitor of the crystallization of uric acid, there were several limitations. In particular, we only examined 20 volunteers, all of whom were healthy non-stone formers, which was reason why uric acid did not crystallize in their urine unless uric acid was added or the sample was acidified. Thus our findings may have limited extrapolation. Another limitation is that urine composition was not the same in the four phases since it is affected by the diet, which was only controlled during the 12 h prior to the collection of samples. Finally, the theobromine sources were cocoa derived products, but the administration of theobromine extracts would be more appropriate, as they would avoid an increase of oxalate urinary concentration in stone-formers.

## 5. Conclusions

Our results indicated that an increased concentration of urinary theobromine reduced the risk of uric acid crystallization in urine. The consumption of moderate amounts of chocolate increased the urinary concentration of theobromine, and this increase depended on the type of chocolate consumed. Considering that chocolate also contains large amounts of sugar and oxalate, it may be more appropriate to administer theobromine supplements, especially for individuals with metabolic syndrome or other pathologies associated with uric acid stones. Further studies of patients with uric acid stones are necessary to prove the effectiveness of theobromine treatment.

## Figures and Tables

**Table 1:** Individual volunteer (*n* = 20) data, mean, median and standard deviation (SD) of urinary parameters (pH, uric acid, oxalate, theobromine, UAC test and uric acid relative supersaturation (RSS) [26]) for basal conditions and under cocoa derived products nutritional intervention.

**Volunteer**	**pH**				**Uric Acid (mg/L)**				**Oxalate (mg/L)**			
1	B	MC	PC	DC	B	MC	PC	DC	B	MC	PC	DC
2	5.85	6.05	5.68	6.39	404	460	461	393	16	17	15	16
3	6.44	6.03	5.54	6.33	346	623	478	555	11	22	10	18
4	7.05	5.73	6.58	5.53	207	142	210	336	37	39	40	34
5	5.00	6.25	6.58	5.72	273	679	398	367	16	25	13	16
6	4.90	5.40	5.04	5.15	453	396	391	525	20	33	19	39
7	5.02	5.60	5.40	5.74	562	709	571	857	37	34	38	58
8	5.35	5.22	5.39	5.10	393	549	156	270	15	29	8	27
9	5.65	5.43	6.35	5.81	326	608	276	516	15	25	10	50
10	5.84	5.56	5.66	5.60	537	558	725	489	36	9	45	26
11	5.54	5.72	6.35	6.60	404	400	317	539	16	39	12	29
12	5.66	5.92	5.68	5.44	469	688	528	405	24	12	28	44
13	6.20	5.62	5.86	5.89	847	281	303	561	31	36	15	23
14	5.33	5.32	5.04	5.53	847	951	389	891	23	24	22	40
15	5.29	5.23	5.15	5.20	728	757	594	610	27	42	31	25
16	5.62	5.49	6.07	5.86	313	113	277	230	23	6	14	18
17	6.15	5.33	6.23	7.03	316	813	283	496	16	24	16	27
18	5.42	5.64	6.22	5.50	392	285	139	508	22	10	10	36
19	6.40	6.58	6.96	5.64	286	720	311	706	24	28	27	42
20	6.06	5.40	5.20	5.53	188	178	252	169	11	10	14	13
mean	5.69	5.65	5.80	5.72	425	507	387	483	22	24	21	32*
median	5.64	5.58	5.68	5.59	393	554	353	502	21	25	16	30
SD	0.56	0.36	0.59	0.48	195	244	167	191	8	11	11	12
**Volunteer**	**Theobromine (mg/L)**				**UAC test**				**Uric acid RSS**			
1	B	MC	PC	DC	B	MC	PC	DC	B	MC	PC	DC
2	8.5	8.5	27.2	51.7	1	2	0	0	−0.37	−0.65	0.15	−1.52
3	0.0	4.2	16.8	20.5	2	4	2	3	−1.76	−0.28	0.51	−1.02
4	2.7	23.2	29.3	32.5	1	1	1	3	−3.47	−1.25	2.58	0.14
5	0.9	9.0	21.0	17.1	4	2	0	1	1.20	−0.64	−1.88	−0.19
6	0.0	4.8	18.3	24.6	1	4	0	0	2.01	0.63	1.49	1.54
7	1.6	5.3	20.5	45.0	3	4	0	1	1.94	0.81	1.03	0.70
8	0.0	6.2	9.4	26.8	2	2	0	1	0.74	1.42	−0.37	0.93
9	2.6	6.7	19.2	37.6	4	3	0	0	−0.16	1.03	−1.83	−0.01
10	0.2	9.1	17.9	28.5	2	2	3	1	−0.03	0.63	0.69	0.40
11	0.0	4.2	15.9	30.3	3	2	1	0	0.32	−0.09	−1.68	−1.58
12	0.0	3.5	15.8	15.6	1	4	0	0	0.22	0.07	0.30	0.56
13	0.0	6.3	15.8	32.7	2	2	1	2	−0.29	−0.26	−0.71	−0.09
14	2.0	20.7	16.2	45.0	6	6	2	4	1.63	1.78	1.48	1.22
15	0.0	4.6	15.2	24.1	6	6	6	4	1.56	1.75	1.67	1.58
16	4.3	1.6	21.8	9.6	2	2	2	1	−0.14	−0.96	−1.25	−1.01
17	0.0	5.7	19.3	36.7	4	4	2	6	−1.27	1.58	−1.56	−2.48
18	0.0	6.1	15.4	37.5	2	2	0	0	0.57	−0.29	−2.32	0.67
19	9.2	10.1	12.9	32.7	2	2	1	1	−1.89	−1.23	−2.86	0.71
20	3.3	7.3	34.0	26.6	1	2	0	1	−1.66	−0.25	0.61	−0.61
mean	2.3	7.6	19.3	30.6	2.6	3.0	1.3	1.6	−0.01	0.18	−0.35	−0.00
median	1.0	6.2	18.1	31.4	2.0	2.0	1.0	1.0	0.09	−0.15	−0.11	0.08
SD	2.4	5.4	5.9	10.3	1.7	1.3	1.7	1.7	1.44	0.97	1.55	1.09

* *p* < 0.05 DC vs. B and PC. B: Basal conditions, MC: Milk chocolate consumption, PC: Cocoa powder consumption, DC: Dark chocolate consumption.

**Table 2:** Uric acid concentration, pH and UAC test reproducibility (*n* = 5) for two different urine samples.

Urine Sample			UAC Test	
	Uric acid (mg/L)	pH	mean	SD
Sample 1	608	5.76	4	0
Sample 2	210	5.69	2	0

SD: Standard deviation of 5 replicates.
